# A latent class approach to understanding patterns of emotional and behavioral problems among early adolescents across four low- and middle-income countries

**DOI:** 10.1017/S0954579422000384

**Published:** 2022-05-30

**Authors:** Shoshanna L. Fine, Robert W. Blum, Judith K. Bass, Aimée M. Lulebo, Anggriyani W. Pinandari, William Stones, Siswanto A. Wilopo, Xiayun Zuo, Rashelle J. Musci

**Affiliations:** 1 Department of Population, Family and Reproductive Health, Johns Hopkins Bloomberg School of Public Health, Baltimore, MD, USA; 2 Department of Mental Health, Johns Hopkins Bloomberg School of Public Health, Baltimore, MD, USA; 3 Department of Epidemiology and Biostatistics, Faculty of Medicine, Kinshasa School of Public Health, University of Kinshasa, Kinshasa, Democratic Republic of Congo; 4 Center for Reproductive Health, Faculty of Medicine, University of Gadjah Mada, Yogyakarta, Indonesia; 5 Center for Reproductive Health, College of Medicine, University of Malawi, Blantyre, Malawi; 6 NHC Key Lab of Reproduction Regulation (Shanghai Institute for Biomedical and Pharmaceutical Technologies), Fudan University, Shanghai, China

**Keywords:** behavioral problems, early adolescents, emotional problems, latent class analysis, low- and middle-income countries, psychosocial development

## Abstract

Early adolescents (ages 10–14) living in low- and middle-income countries have heightened vulnerability to psychosocial risks, but available evidence from these settings is limited. This study used data from the Global Early Adolescent Study to characterize prototypical patterns of emotional and behavioral problems among 10,437 early adolescents (51% female) living in the Democratic Republic of Congo (DRC), Malawi, Indonesia, and China, and explore the extent to which these patterns varied by country and sex. LCA was used to identify and classify patterns of emotional and behavioral problems separately by country. Within each country, measurement invariance by sex was evaluated. LCA supported a four-class solution in DRC, Malawi, and Indonesia, and a three-class solution in China. Across countries, early adolescents fell into the following subgroups: Well-Adjusted (40–62%), Emotional Problems (14–29%), Behavioral Problems (15–22%; not present in China), and Maladjusted (4–15%). Despite the consistency of these patterns, there were notable contextual differences. Further, tests of measurement invariance indicated that the prevalence and nature of these classes differed by sex. Findings can be used to support the tailoring of interventions targeting psychosocial adjustment, and suggest that such programs may have utility across diverse cross-national settings.

Early adolescence (ages 10–14) is a critical developmental period, with the social–emotional skills and health-related behaviors that emerge during this time serving as a foundation for future well-being (McCarthy et al., 2016; Sawyer et al., 2012). With a substantial proportion of lifetime mental health problems manifesting by age 14 (Kessler et al., 2005), poor psychosocial adjustment during early adolescence can set the stage for impairment throughout the life course (Patel et al., 2008; Patton et al., 2016). While approximately 90% of the world’s adolescents live in low- and middle-income countries (LMICs) (UNICEF, 2012), only a fraction of the research on adolescent psychosocial development has been conducted in these settings (Patel et al., 2008), and even less has focused specifically on early adolescent populations (McCarthy et al., 2016). This represents a serious gap in evidence, as early adolescents living in LMICs are disproportionately vulnerable due to factors such as forced displacement, migration, violence, socioeconomic deprivation, and gender inequality (Fatusi & Hindin, 2010).

The nature of early adolescence as a sensitive period for psychosocial development underscores the importance of improving strategies for identifying youth at risk of experiencing long-term adjustment issues. Longitudinal studies conducted in high-income countries have identified a number of emotional and behavioral indicators during adolescence that are predictive of negative outcomes later in life. For instance, two recent systematic reviews found that depressive symptoms in adolescence increase the likelihood of adult mental health problems (Johnson et al., 2018), and are also associated with low educational attainment and unemployment (Clayborne et al., 2019). Similarly, early symptoms of anxiety have been linked with subsequent anxiety, depression, suicidality, and harmful substance use (Doering et al., 2019; Dyer et al., 2019). In addition, adolescent behavioral problems, including involvement in interpersonal aggression as a victim or perpetrator (Copeland et al., 2013; Gibb et al., 2011; Sigurdson et al., 2015) and drug and alcohol use (Gobbi et al., 2019; Silins et al., 2018; Wilkinson et al., 2016), are predictive of a range of negative mental health and psychosocial outcomes in adulthood.

While studies focused on individual psychosocial risks in adolescence have utility in uncovering etiologic pathways for specific mental disorders, such approaches overlook the common co-occurrence of emotional and behavioral problems during this developmental period. A substantial body of literature has found that these problems are rarely found in isolation: adolescents with emotional distress often exhibit symptoms of both depression and anxiety (Cummings et al., 2014; Melton et al., 2016); those involved in interpersonal aggression frequently report victimization as well as perpetration experiences (Haynie et al., 2001; Nansel et al., 2001); behavioral problems, including aggression and substance use, typically cluster together (Doran et al., 2012; Luk et al., 2012); and emotional problems may be found alongside behavioral ones (Angold et al., 1999; Boylan et al., 2007). A range of theoretical models have been used to explain these complex patterns of psychosocial development. For example, Problem Behavior Theory suggests that adolescent risk behaviors frequently co-occur due to a single underlying dimension of psychosocial vulnerability (Jessor & Jessor, 1977); the Self-Medication Hypothesis theorizes that individuals frequently use substances as a means of coping with emotional distress (Khantzian, 1997); researchers have argued that adolescent depression and anxiety are separate but highly related constructs with multiple pathways leading to their co-occurrence (Cummings et al., 2014); and emerging evidence has found a “general psychopathology factor” underlying both emotional and behavioral problems among adolescents (Patalay et al., 2015). Importantly, regardless of the specific theoretical model, research has generally agreed that such co-occurrence confers additional vulnerability for adolescents (Angold et al., 1999; Copeland et al., 2013; Lewinsohn et al., 1995).

In order to address these complexities, person-centered statistical approaches such as LCA and LPA are increasingly used to examine heterogeneity in psychosocial development among adolescent populations (Lanza & Cooper, 2016). These approaches identify distinct subgroups (i.e., classes) of individuals who are similar to each other but different from members of other subgroups based on their patterns of endorsement across a set of indicator variables, with LCA used for categorical indicators and LPA used for continuous indicators (Collins & Lanza, 2010). By allowing for this type of categorization, person-centered approaches stand in contrast to traditional variable-centered approaches such as factor analysis, which are most appropriate when an underlying latent construct is dimensional in nature (Ruscio & Ruscio, 2008). In the context of adolescent psychosocial development, LCA/LPA can be used to identify subgroups of adolescents who share similar patterns of emotional and behavioral problems, which can ultimately aid in targeting those who may particularly benefit from early intervention efforts (Lanza & Rhoades, 2013; Nylund-Gibson & Hart, 2014). In addition, these methods have broad utility in cross-cultural research, as they allow for flexible comparisons of model similarities and differences across diverse populations (Kankaraš et al., 2010). As such, these methods are well-suited for extending a substantial body of factor analytic work exploring the cross-national structure of emergent psychopathological syndromes among children and adolescents (Ivanova et al., 2007; Rescorla et al., 2007, 2012).

While a growing number of studies have used LCA/LPA to examine patterns of psychosocial risks among adolescents living in high-income countries (e.g., Bianchi et al., 2017; Eastman et al., 2018; Kretschmer et al., 2015; Luk et al., 2012), few have applied these methods to those living in LMICs (e.g., Abbasi-Ghahramanloo et al., 2018; González-Forteza et al., 2017; Ma et al., 2019; Zhao et al., 2019). Further, studies have generally been restricted to either emotional or behavioral problems rather than simultaneously examining a broader spectrum of psychosocial risks, and have rarely focused exclusively on early adolescent populations. Existing studies that *have* investigated co-occurring emotional and behavioral problems among early adolescents have uncovered strikingly similar results. For instance, two studies from Italy (Bianchi et al., 2017; *N* = 3418) and the Netherlands (Kretschmer et al., 2015; *N* = 2149) used person-centered approaches to classify early adolescents based on an analogous set of internalizing (e.g., depression, anxiety, somatic complaints) and externalizing (e.g., rule-breaking, aggression) symptoms, and uncovered four distinct subgroups: an internalizing class, an externalizing class, a low problem normative class, and a comorbid problem dysfunctional class.

An additional limitation of the extant literature is the lack of multicountry studies which employ person-centered approaches to assess the contextual generalizability of developmental subgroups in adolescence. Nearly all existing analyses have been conducted within a single population, precluding an understanding of whether patterns of psychosocial risks differ in meaningful ways across diverse settings. Further, as many existing studies have been implemented among subpopulations with specific vulnerabilities (e.g., criminal-justice-involved youth, youth diagnosed with a mental disorder, substance users) (Dembo et al., 2012; Vaughn et al., 2012; Williams et al., 2013), findings may have limited general applicability. One notable exception is Jordan et al. (2016), who used LCA among 38,070 adolescents from population-based samples in 34 societies to determine the cross-cultural existence and prevalence of youth belonging to a “dysregulation profile,” characterized by co-occurring internalizing symptoms, attention issues, and aggressive behaviors. While the authors found some evidence that this subgroup exists across different environments, there was substantial variation in their results. Further, Ethiopia was the only low-income country included in their analysis.

Finally, none of these studies has investigated potential sex differences within psychosocial risk subgroups through an examination of sex-related measurement invariance. In the context of LCA/LPA, measurement invariance holds if individuals from different subpopulations (e.g., boys and girls) but within the same class have identical patterns of endorsement across the included indicator variables; a lack of measurement invariance is an indication of qualitative differences in the interpretation of classes between subpopulations (Collins & Lanza, 2010). It is well-established that psychosocial risks differ between adolescent girls and boys, with girls generally demonstrating more emotional and boys more behavioral problems (Zahn-Waxler et al., 2015). While most existing studies using LCA/LPA have addressed these discrepancies by including sex as a covariate in their analyses, this approach can only account for sex differences in the likelihood of class membership; it cannot uncover important interpretive distinctions. Ignoring these differences can result in model misspecification and biased scientific conclusions (Collins & Lanza, 2010; Masyn, 2017); as such, there is a need for studies that formally evaluate measurement invariance by sex when considering psychosocial risk profiles.

The current study seeks to address these gaps by investigating variations in psychosocial development among early adolescents living in four LMICs across three continents. LCA was used to identify and characterize prototypical patterns of emotional and behavioral problems among 10- to 14-year-olds from six low-income urban settings in the Democratic Republic of Congo (DRC), Malawi, Indonesia, and China. The study had two primary aims: (a) to explore similarities and differences in psychosocial risk patterns across countries; and (b) to determine the extent to which these patterns varied between boys and girls within each country. While this investigation was exploratory in nature, we hypothesized that we would find similar psychosocial risk subgroups among early adolescents as those uncovered in high-income country contexts (Bianchi et al., 2017; Kretschmer et al., 2015). Given developmental differences between boys and girls within this age group, however, we hypothesized that there would be significant measurement noninvariance by sex.

## Method

### Participants

Participants were drawn from the Global Early Adolescent Study (GEAS), an international collaboration between the Johns Hopkins Bloomberg School of Public Health (BSPH), the World Health Organization (WHO) and research institutions in participating countries (Mmari et al., 2021). The GEAS is a longitudinal study which seeks to understand risk and protective factors for healthy development among early adolescents living in low-resource urban settings around the world. The current study used cross-sectional baseline data from Kinshasa, DRC (*n* = 2006; 51.5% girls); Blantyre, Malawi (*n* = 2016; 49.6% girls); Semarang, Bandar Lampung, and Denpasar, Indonesia (*n* = 4657; 53.0% girls); and Shanghai, China (*n* = 1758; 48.6% girls). The analytic sample of 10,437 adolescents excluded 33 participants who were missing data across all of the emotional and behavioral problems.

The four included countries were selected from among those participating in the GEAS in order to compare LMICs with diverse cultural, economic, and geographic environments. DRC and Malawi are among the poorest countries in the world, with 76.6% and 70.3% of the population living in extreme poverty, respectively (UNDP, 2019). Both Kinshasa and Blantyre have experienced rapid urbanization in recent years due in part to limited economic prospects in rural areas, as well as conflict-related migration in DRC (Choi et al., 2016; World Bank, 2018). By contrast, China is the world’s second largest economy, a status it has achieved through four decades of rapid economic growth and social transformation (WHO, 2016). Working class families in Shanghai, however, may not experience the benefits of urbanization, with particularly marked disparities among rural-to-urban migrants (World Bank, 2014). Like China, Indonesia has enjoyed notable economic development over the past several decades; however, this development has been accompanied by rising interregional disparities in health, education, and income which may be especially pronounced in the country’s urban areas (WHO, 2017). While it is a majority Muslim country, Indonesia is characterized by immense ethnic, cultural, and linguistic diversity: for instance, of the three included cities, Bandar Lampung has a more conservative Muslim population than Semarang, whereas Denpasar has a majority Hindu population (Wilopo et al., 2020).

### Procedures

Detailed site-specific study procedures for the GEAS have been described elsewhere (Mmari et al., 2021). In brief, early adolescents were sampled from participating schools in each country. These schools were purposively selected to target students living in low-resource urban areas, and included 66 schools in Kinshasa; 4 schools Blantyre; 6 schools each in Semarang, Bandar Lampung, and Denpasar; and 3 schools in Shanghai. Eligible adolescents from each school were recruited by the country’s research team in collaboration with school personnel. Prior to data collection, informed consent was obtained from adolescents’ primary caregivers and assent was obtained from adolescents. Ethical approval was given by the Institutional Review Board (IRB) of the primary research institution in each participating country, as well as the BSPH IRB.

Data collection took place at each participating school during or after regular school hours. Questionnaires were largely self-administered via mobile tablets through the use of computer-assisted self-interview (CASI) for increased privacy. For participants with low literacy, trained data collectors administered questionnaires through the use of computer-assisted personal interview (CAPI). In DRC and Indonesia, primary caregivers were also interviewed in the same manner in order to provide sociodemographic and household information. Cross-sectional baseline data collection was completed between 2017 and 2018.

### Measures

Each study country used a standardized assessment instrument containing information on domains relevant to adolescent development, including mental health, substance use, and interpersonal aggression. This instrument was developed during a 3-year formative study which used a mixed methods approach to formulate a set of cross-culturally appropriate questions for assessing key domains of health and development among early adolescents living in diverse settings. Prior to data collection, the instrument was translated into the local language(s) in each country, and back-translated by separate translators to ensure comparability of meaning. It then underwent two phases of pilot-testing: first, among 1944 adolescents in 14 countries, and after revision, among 434 adolescents in 6 countries. This extensive process yielded a final instrument expected to have applicability across extremely varied samples. Further details regarding instrument development and validation have been published previously (Blum, Li, & Naranjo-Rivera, 2019; Mmari et al., 2017; Moreau et al., 2019; Zimmerman et al., 2019), and the standardized assessment instrument is available from www.geastudy.org.

### Emotional problems

Emotional problems were measured using five indicators capturing symptoms of depression and anxiety: (a) “I blame myself when things go wrong,” (b) “I worry for no good reason,” (c) “I am so unhappy I can’t sleep at night,” (d) “I feel sad,” and (e) “I am so unhappy I think of harming myself.” Adolescents rated how much they agreed with each item on a five-point scale, where response options included “agree a lot,” “agree a little,” “neither agree nor disagree,” “disagree a little,” and “disagree a lot.” In order to increase interpretability of results, support model parsimony, and facilitate simultaneous analysis alongside dichotomous behavioral problem indicators, all emotional problem indicators were dichotomized, with those who agreed a little or a lot coded as positively endorsing the symptom.

### Behavioral problems

Behavioral problems were measured using five indicators capturing interpersonal aggression and substance use. Two indicators assessed past-six month perpetration of interpersonal aggression: (a) “Bullied or threatened another boy or girl for any reason,” and (b) “Slapped, hit, or otherwise physically hurt another boy or girl.” Two assessed past-6 month experiences of peer victimization: (a) “Been teased or called names by someone,” and (b) “Been slapped, hit, or otherwise physically hurt by a boy or girl.” Both perpetration and victimization were included as indicators of behavioral problems given strong prior evidence that these experiences are often inextricably linked (Haynie et al., 2001; Nansel et al., 2001) and together may represent an important marker of psychosocial maladjustment (Giang & Graham, 2008; Schwartz et al., 2001). Finally, one indicator captured lifetime use of one or more substances, including alcohol, tobacco, marijuana, and/or illicit drugs. While questions assessed each of these substances individually, they were considered collectively for the purposes of this analysis for two primary reasons: (a) any substance use, regardless of the specific type, was hypothesized to be meaningful given the young age of participants; and (b) cross-cultural differences related the use of specific substances (e.g., alcohol use in the predominately Muslim country of Indonesia). Across all of the behavioral problem indicators, response options included “yes” or “no.”

### Sociodemographic characteristics

The assessment instrument also collected basic sociodemographic information including age, household size, migration status (born outside of the current city or not), primary caregiver (mother, father, grandparent, or other), primary caregiver’s marital status (married/living together or unmarried/separated/widowed), primary caregiver’s education (completed primary school or less, completed some or all secondary school, or completed some or all vocational school/university), and primary caregiver’s employment status (employed/retired or unemployed). In DRC and Indonesia, primary caregiver-reported information included household size, marital status, education, and employment status. In Malawi, migration status as well as primary caregiver’s marital status, education, and employment status were not reported.

### Data analysis

LCA was conducted within each country to identify participants with similar patterns of responses on included psychosocial risk indicators. This type of model produces two sets of parameters: (a) latent class probabilities, which reflect the prevalence of each class; and (b) item-response probabilities, which represent the probability of endorsing a particular indicator given membership in a class. The procedure for conducting LCA involves class enumeration, whereby models are tested with an increasing number of classes, and fit indices are compared to determine the best fitting model (Collins & Lanza, 2010). Model fit was evaluated using a number of fit indices, including the Akaike Information Criterion (AIC) (Akaike, 1987), Bayesian Information Criterion (BIC) (Schwarz, 1978), sample-size adjusted BIC (aBIC) (Sclove, 1987), Vuong-Lo-Mendell-Rubin Likelihood Ratio Test (VLMR) (Lo et al., 2001), and bootstrapped likelihood ratio test (BLRT) (McLachlan & Peel, 2000). Particular weight was given to the BIC, as prior simulations have found it to be among the most accurate in suggesting the appropriate number of classes (Nylund et al., 2007). Goodness of fit was further assessed through entropy scores, which indicate classification quality. Finally, the theoretical interpretability of classes was considered. Across all models, missing indicator data was addressed through the use of full information maximum likelihood estimation (Muthén & Shedden, 1999). The rates of missing values across countries were no more than 5% for the emotional problem indicators and no more than 11% for the behavioral problem indicators. Due to the multilevel data structure, with adolescents nested within schools, standard errors were adjusted for clustering through the use of sandwich estimators. As this adjustment makes the VLMR and BLRT uninterpretable, however, models were rerun without clustered standard errors for the purposes of fit evaluation. All analyses were performed in *Mplus* version 8.1.6 (Muthén & Muthén, 1998–2017).

Following initial class enumeration procedures, measurement invariance by sex was evaluated within each country using a multiple-group approach (Collins & Lanza, 2010; Kankaraš et al., 2010). First, class enumeration procedures were performed separately for boys and girls within each country in order to establish whether the general latent structure (i.e., number of classes) was similar across sex. Second, an omnibus test of measurement invariance was conducted, comparing a model in which all parameters (i.e., latent class probabilities and item-response probabilities) were allowed to vary by sex to a model in which item-response probabilities were constrained to be equal for boys and girls. Assuming evidence for significant measurement variance, a series of nested models were then tested comparing the fully unconstrained model to a model in which one indicator at a time was constrained to be equal across groups. The logic behind this stepwise approach is that it allows for the identification of specific indicators with differential functioning by sex (Masyn, 2017). Next, the fully unconstrained model was compared to a series of models in which multiple indicators with reasonable evidence for measurement invariance were constrained to be equal for boys and girls. Lastly, the final partially invariant model was compared to an equivalent model in which latent class probabilities were also constrained to be equal across groups. All nested models were compared using likelihood-ratio tests (*G*^2^_Δ_).

## Results

### Sample characteristics

Sociodemographic characteristics for the sample are presented in Table 1, alongside descriptive statistics for each of the psychosocial risk indicators. The average age of participants was roughly comparable, ranging from 11.9 (*SD* = 1.4) years old in DRC to 12.5 (*SD* = 1.0) years old in China. The majority of participants in three countries listed their mother as their primary caregiver: Malawi (73.7%), Indonesia (70.2%), and China (70.9%); in DRC, the father was the primary caregiver for 57.0% of respondents. Across DRC, Indonesia, and China, the majority of primary caregivers had attended at least secondary school (DRC: 89.1%; Indonesia: 81.7%; China: 82.7%), and most were employed or retired (DRC: 75.6%; Indonesia: 58.0%; China: 85.0%). Among the same countries, China had the highest levels of migration, with 15.0% of participants reporting that they had been born outside of Shanghai.

The prevalence of emotional and behavioral problems varied widely across countries. Among the symptoms of depression and anxiety, the highest reported prevalences were for *self-blame*, which ranged from 60.8% in Indonesia to 75.2% in China, and the lowest were for *thinking of self-harm*, which ranged from 4.7% in DRC to 29.6% in Malawi. For emotional problems other than *self-blame*, among the study countries DRC consistently had the lowest prevalences and Malawi the highest. For aggressive behaviors and peer victimization, China had the lowest prevalences, ranging from 4.4% for *slapping/hitting/hurting* to 31.8% for *being teased/called names*. Malawi had the highest prevalences for the same indicators, ranging from 24.1% for *bullying/threatening* to 52.7% for *being teased/called names*. The prevalence of lifetime *substance use* ranged from 10.4% in DRC to 27.0% in China. It is important to note that these reported prevalences are based on dichotomized indicators and thus represent the presence or absence of psychosocial risk rather than clinical significance.

### Initial class enumeration

A series of latent class models ranging from one to seven classes were estimated within each country. Fit indices used for model selection are presented in Table 2. In DRC, the BIC and aBIC both supported a four-class solution; in Malawi, they supported five- and six-class solutions, respectively; in Indonesia, they supported a seven-class solution; and in China, they supported three- and four-class solutions. While the VLMR did not indicate the best-fitting model in China or Indonesia, it favored a four-class solution in both DRC and Malawi, as this was the greatest number of classes for which the test remained statistically significant. This suggests that in DRC and Malawi, the four-class model significantly improved fit over the three-class model, but the five-class model did not improve fit over the four-class model. The BLRT was not informative for model selection in any of the countries. Given that all of these fit statistics are sensitive to sample size (Collins & Lanza, 2010), the Indonesia data was rerun separately by city (i.e., Semarang, Bandar Lampung, and Denpasar) to provide further information on an appropriate solution: fit indices suggested that a four-, five-, or six-class model would be acceptable (Supplemental Table 1). An examination of the plotted BIC and aBIC values in each city revealed a plateau in values after the four-class model in each case, indicating that improvements in model fit from adding additional classes were relatively insubstantial. Importantly, there were no substantive differences in parameters between the four-class models in each city, supporting the appropriateness of considering these samples together. Finally, an investigation of item-response and latent class probability patterns showed that four-class solutions were clearly interpretable across DRC, Malawi, and Indonesia, and that they also had acceptable classification quality, with entropy ≥0.72. In China, however, the four-class solution was marked by estimability issues, as it resulted in one class with a low class prevalence (3%) and several bounded item-response probabilities (i.e., probability = 1.00). Taking the above criteria as a whole, and in the interest of model parsimony and substantive interpretability, a four-class model was selected as being appropriate in DRC, Malawi, and Indonesia, and a three-class model was selected as being appropriate in China.

### Initial country-specific class descriptions

**Figure 1.**
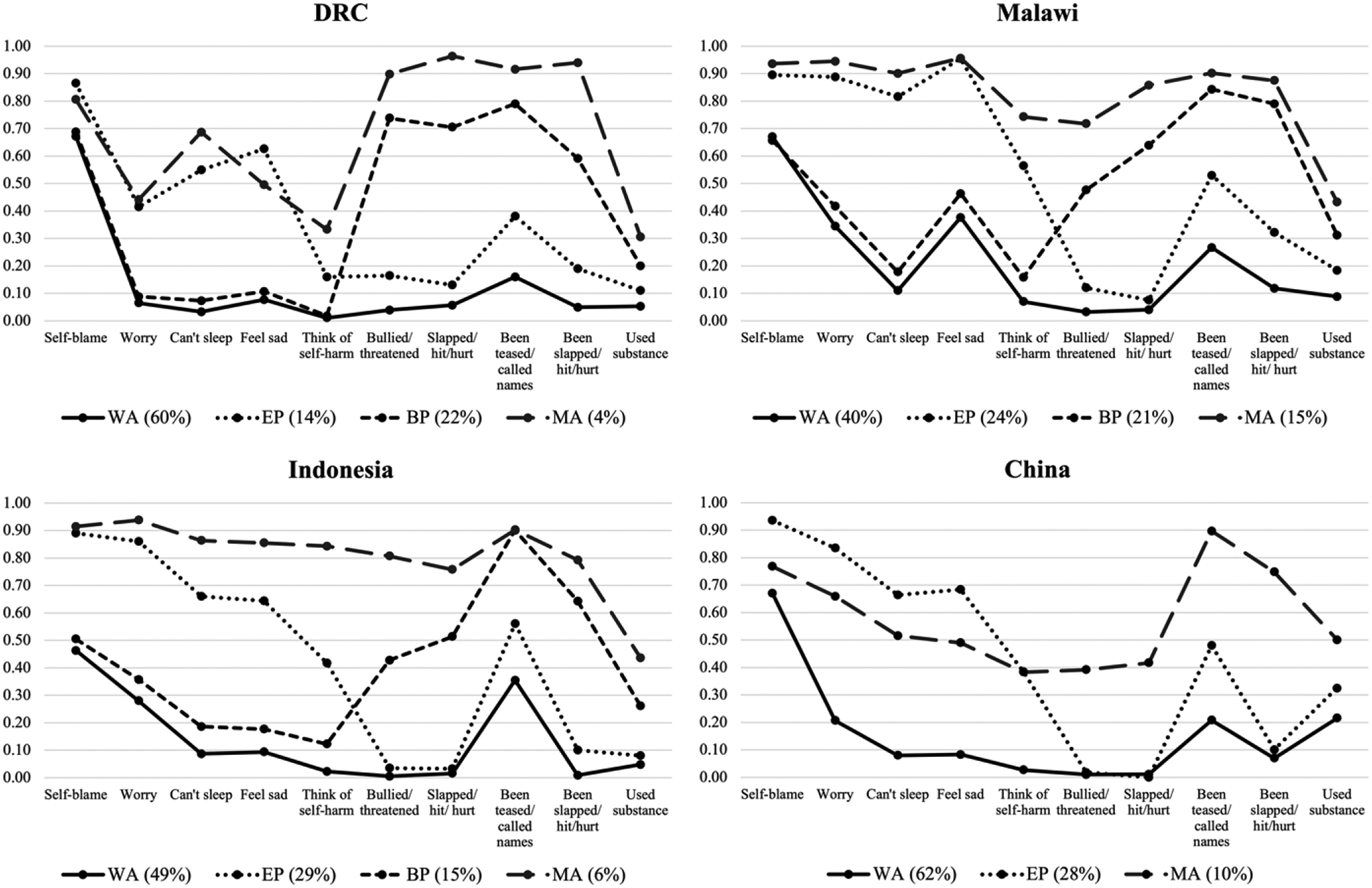
Estimated item-response probabilities for the latent class models in each country. *Note*. WA = Well-Adjusted; EP = Emotional Problems; BP = Behavioral Problems; MA = Maladjusted.

Parameter estimates for the latent class models in each country, including latent class prevalences and item-response probabilities, are illustrated in Figure 1. While there were some cross-country variations, four relatively consistent patterns emerged among early adolescents. Most adolescents were in the *Well-Adjusted* class (DRC: 60%; Malawi: 40%; Indonesia: 49%; China: 62%), which included those with a low likelihood (item-response probabilities <0.25) of endorsing almost all of the emotional and behavioral problems. The greatest exception to this was *self-blame*, which had much higher item-response probabilities (0.46–0.67) across all of the countries. In addition, adolescents in this class in Malawi and Indonesia had a somewhat higher likelihood of endorsing *worrying* (0.35 and 0.28, respectively) and *being teased/called names* (0.27 and 0.36) compared to those in DRC and China. Conversely, adolescents in this class in China had a moderately elevated probability of endorsing substance use (0.22).

Adolescents in the *Emotional Problems* class (DRC: 14%; Malawi: 24%; Indonesia: 29%; China: 28%) were generally likely (item-response probabilities >0.50) to endorse emotional but not behavioral problems. While *thinking of self-harm* was elevated among adolescents in this class, item-response probabilities across all of the countries were lower than those of the other depressive and anxiety symptoms (0.16–0.57). In addition, across all of the countries, adolescents in this class had a moderate likelihood of endorsing *being teased/called names* (0.38–0.56).

While the *Behavioral Problems* class did not emerge in China, adolescents in this class across the other three countries (DRC: 22%; Malawi: 21%; Indonesia: 15%) were generally likely (item-response probabilities >0.50) to endorse behavioral but not emotional problems. As in the *Well-Adjusted* class, the exception to this was *self-blame*, which had elevated item-response probabilities (0.51–0.69) across the three included countries. In addition, the probabilities of endorsing *substance use* among adolescents in this class were somewhat lower than the other behavioral problems (0.20–0.31).

The least prevalent class across countries was the *Maladjusted* class (DRC: 4%; Malawi: 15%; Indonesia: 6%; China: 10%), which included those with a high likelihood (item response probabilities >0.50) of endorsing almost all of the emotional *and* behavioral problems. This class had some of the most marked cross-national differences. Specifically, adolescents in this class in DRC and China had a lower likelihood of endorsing all of the emotional problems other than *self-blame* (item-response probabilities of 0.33–0.69, compared to those ≥0.74 in Malawi and Indonesia). In addition, adolescents in this class in China had a lower likelihood of endorsing both aggression indicators (item-response probabilities of 0.39–0.42, compared to those ≥0.72 in the other countries). By contrast, the probabilities of endorsing *substance use* were higher among adolescents in China (item-response probability of 0.50, compared to 0.31–0.44 in the other countries).

### Sex-specific measurement invariance testing

Prior to formal measurement invariance testing, sex-specific latent class models ranging from one to six classes were estimated within each country (Supplemental Table 2). Class enumeration procedures confirmed that four-class solutions were appropriate for both boys and girls in DRC, Malawi, and Indonesia, and three-class solutions were appropriate for both boys and girls in China.

**Figure 2.**
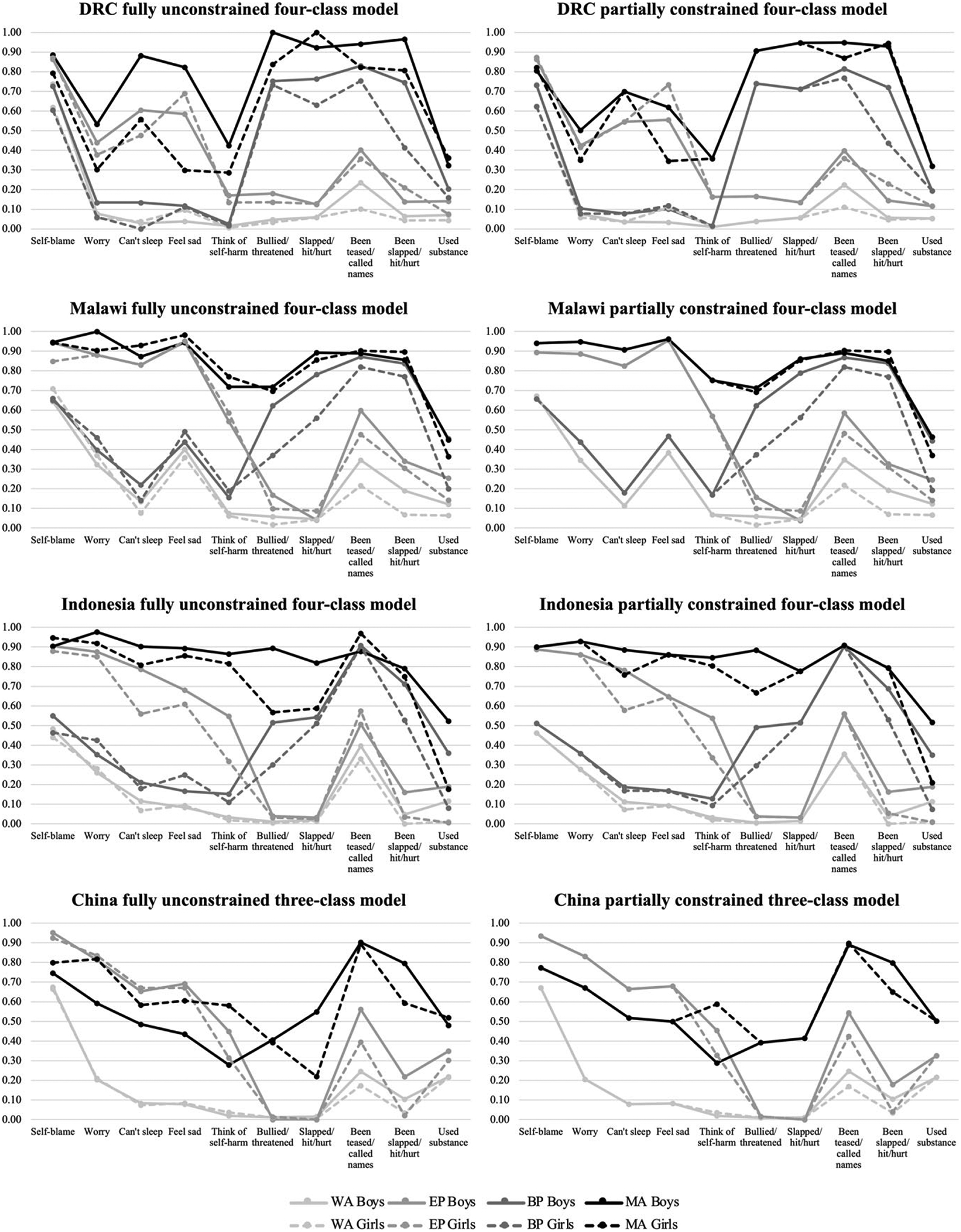
Estimated item-response probabilities for the fully unconstrained and partially constrained multigroup latent class models in each country. *Note*. WA = Well-Adjusted; EP = Emotional Problems; BP = Behavioral Problems; MA = Maladjusted.

Results from the comparisons of nested multigroup models in each country are laid out in Table 3, and parameter estimates for the fully unconstrained models in each country are represented in Figure 2. Across countries, omnibus tests of measurement invariance by sex were highly significant, indicating that constraining the item-response probabilities to be equal for boys and girls decreased model fit relative to the fully unconstrained model (DRC: *G*^2^_Δ_ = 119.37, df = 40, *p* < 0.001; Malawi: *G*^2^_Δ_ = 102.36, df = 40, *p* < 0.001; Indonesia: *G*^2^_Δ_ = 386.82, df = 40, *p* < 0.001; China: *G*^2^_Δ_ = 78.86, df = 30, *p* < 0.001). Testing for differential functioning of individual indicators among boys and girls yielded mixed results in each country. In DRC, there was evidence for measurement invariance of the indicators capturing *sleeplessness*, *thinking of self-harm*, *bullying/threatening*, *slapping/hitting/hurting*, and *substance use*. In Malawi, there was evidence for measurement invariance of the indicators capturing *feeling sad* and *thinking of self-harm*; in addition, an examination of the fully unconstrained four-class model revealed only minor differences between boys and girls in the item-response probabilities for *self-blame*, *worrying*, and *sleeplessness* across classes. In Indonesia, there was evidence for measurement invariance of the indicators capturing *self-blame*, *worrying*, *feeling sad*, *slapping/hitting/hurting*, and *being teased/called names*. In China, there was evidence for measurement invariance of the indicators capturing *self-blame*, *sleeplessness*, *bullying/threatening*, *slapping/hitting/hurting*, and *substance use*; and further inspection revealed few substantive differences in the item-response probabilities for *worrying and feeling sad* across classes

Following this initial process, partially invariant models were specified in each country which constrained the item-response probabilities of the above-mentioned indicators; formal tests of these models indicated no significant differences in model fit from the fully unconstrained model (DRC: *G*^2^_Δ_ = 23.89, df = 20, *p* = 0.247; Malawi = 28.28, df = 20, *p* = 0.103; Indonesia: *G*^2^_Δ_ = 31.15, df = 20, *p* = 0.053; China: *G*^2^_Δ_ = 25.72, df = 21, *p* = 0.217). As a final step, these partially invariant models were compared to equivalent models in which latent class probabilities were also constrained; results showed that this significantly decreased model fit in each country. As such, partially invariant models were selected as the final models across study countries as these models were more parsimonious than the fully unconstrained models while still allowing specific indicators with significant differential functioning to vary by sex.

### Final model results

Parameter estimates for the final sex-specific models in each country are illustrated in Figure 2. While the same patterned subgroups emerged among boys and girls as outlined above, there were sex differences between equivalent classes within each country.

### DRC

In DRC, boys were more likely than girls to be in all of the high-risk classes, with 16% of boys compared to 12% of girls in the *Emotional Problems* class, 25% of boys compared to 18% of girls in the *Behavioral Problems* class, and 5% of boys compared to 3% of girls in the *Maladjusted* class. Girls in the *Maladjusted* class were less likely than their male counterparts to endorse *worrying* and *feeling sad*, and those in the *Behavioral Problems* class were less likely to endorse *being slapped/hit/hurt*; those in the *Emotional Problems* class were more likely to endorse *feeling sad*.

### Malawi

In Malawi, boys were more likely to be in the *Maladjusted* class (16% boys, 14% girls), but girls were more likely to be in the *Emotional Problems* (21% boys, 27% girls) and *Behavioral Problems* (19% boys, 21% girls) classes. Girls in the *Behavioral Problems* class were less likely to endorse *bullying/threatening*, *slapping/hitting/hurting*, and *substance use*.

### Indonesia

In Indonesia, boys were more likely to be in the *Behavioral Problems* (21% boys, 10% girls) and *Maladjusted* (9% boys, 4% girls) classes, whereas girls were more likely to be in the *Emotional Problems* class (25% boys, 33% girls). Boys in Indonesia had a substantially higher likelihood of reporting lifetime *substance use* across all of the classes, as well as *sleeplessness* in the *Emotional Problems* and *Maladjusted* classes, *bullying/threatening* in the *Behavioral Problems* and *Maladjusted* classes, *thinking of self-harm* in the *Emotional Problems* class, and *being slapped/hit/hurt* in the *Behavioral Problems* class.

### China

In China, boys were more likely to be in the *Maladjusted* class (13% boys, 7% girls), but girls were more likely to be in the *Emotional Problems* class (23% boys, 33% girls). Girls in the *Maladjusted* class were more likely to endorse *thinking of self-harm*, and less likely to endorse *being slapped/hit/hurt*. Boys in the *Emotional Problems* class had a higher likelihood of reporting *thinking of self-harm*, *being teased/called names*, and *being slapped/hit/hurt*.

## Discussion

The current study used a person-centered analytic approach to investigate variations in psychosocial development during early adolescence (ages 10–14), with a specific focus on youth living in low-resource urban settings in DRC, Malawi, Indonesia, and China. Despite immense cultural and contextual variability across the four study countries, we found striking similarities in patterns of emotional and behavioral problems. Results suggested the presence of four general subgroups: a *Well-Adjusted* class, with very few problems; an *Emotional Problems* class, with heightened symptoms of depression and anxiety; a *Behavioral Problems* class (not present in China), with elevated involvement in aggressive behaviors, peer victimization, and substance use; and a *Maladjusted* class, with co-occurring emotional and behavioral problems. These findings align with prior research conducted in Italy and the Netherlands, which uncovered very similar four-class results among community samples of early adolescents (Bianchi et al., 2017; Kretschmer et al., 2015); equivalent subgroups have also emerged in studies including older adolescents in the United States, New Zealand, and China (Bonadio et al., 2016; Ma et al., 2019; Noel et al., 2013). Notably, a recent systemic review of studies utilizing LCA to investigate patterns of mental health problems in children found that for those examining both emotional and behavioral problems, the most common outcome was a four-class solution including asymptomatic, purely emotional, purely behavioral, and comorbid classes (Petersen et al., 2019). Together, these findings suggest that not only are these psychosocial risk patterns widespread among early adolescents across diverse global settings – including low-, middle-, and high-income country contexts – but that they also may have stability from childhood through adolescence.

A further notable finding relates to the likely presence of a *Maladjusted* class (4–15%) across countries, characterized by elevated probabilities of endorsing depressive symptoms, anxiety symptoms, aggressive behaviors, peer victimization, and substance use. This aligns with the work of Althoff et al. (2010), who first utilized the term “dysregulation profile” to describe a subgroup of children and adolescents with co-occurring internalizing symptoms, attention issues, and aggressive behaviors. Prior research on the dysregulation profile has found evidence for its existence among adolescents in a diverse set of countries around the globe, including a number of LMICs, with prevalences varying between 1% and 26% in community samples (Jordan et al., 2016; Rescorla et al., 2020). While we included a somewhat different set of emotional and behavioral indicators than those traditionally comprising the dysregulation profile – most notably the lack of attention problems – our findings lend support for the presence of this subgroup among adolescents living in situations of adversity worldwide. Moreover, our inclusion of a wider range of psychosocial indicators – including those related to bullying and substance use – aligns with prior studies suggesting that adolescents who exhibit dysregulation are likely to do so across multiple emotional and behavioral domains (Biederman et al., 2012; Deutz et al., 2016; Haltigan et al., 2018; Masi et al., 2015). This has particular relevance to early intervention efforts, as it suggests that adolescents who fall within this vulnerable subgroup may especially benefit from targeted services meeting both their emotional and behavioral needs.

Despite the overall consistency in patterns of emotional and behavioral problems across countries, we uncovered several important differences that merit further discussion. In particular, the lack of a clear *Behavioral Problems* class in China is noteworthy, as it speaks to the influence of cultural factors in the expression of psychosocial risk among early adolescents. In China, we hypothesize that traditional cultural values around social harmony inhibit the development and expression of aggressive and delinquent behaviors, diminishing the likelihood that youth without any underlying emotional issues would engage in such behaviors (Chen, 2010). To note, two prior studies have utilized LPA to examine co-occurring emotional and behavioral problems among Chinese adolescents: one focused on *left-behind* adolescents in rural eastern China (Zhao et al., 2019), and one comparing Tibetan and Han adolescents in the north (Ma et al., 2019). In both studies, a subgroup emerged that was characterized by heightened behavioral issues (e.g., aggressive, rule-breaking, and antisocial behaviors), which the authors labeled as an “externalizing problems” profile. In both cases, however, adolescents belonging to this subgroup *also* had moderate-to-high levels of emotional issues (e.g., depressive symptoms, loneliness, and negative affect); as such, they more closely resemble those that we have classified as “maladjusted.” This strengthens our hypothesis that there may be unique factors in China which diminish the likelihood of a purely behavioral class among adolescents.

A further difference is present in the country-specific nuances within these latent classes. In particular, while the overall class structures were relatively consistent across countries, the conditional probabilities of emotional and behavioral indicators within equivalent classes demonstrated marked variability. This manifested most strongly in the *Maladjusted* class, which had comparatively lower probabilities of depressive and anxiety symptoms in DRC and China, and lower endorsement of aggressive behaviors in China. Likewise, in the *Well-Adjusted* class, there was notably higher endorsement of substance use in China compared to the other countries, and there was somewhat elevated endorsement of *worrying* and *being teased/called names* in Malawi and Indonesia. While such results can partially be explained by empirical differences between samples – for instance, the lower overall prevalence of reported emotional problems in the DRC sample – they are again suggestive of the role that cultural factors can play in the manifestation of psychosocial risk. For example, prior research has found the use of alcohol to be relatively normative among Chinese youth, with high rates of alcohol initiation before the age of 13 (Feng & Newman, 2016). Qualitative studies have suggested that moderate drinking in social settings is widely accepted among adolescents (Yoon et al., 2015), and may be facilitated by a traditional drinking culture that values alcohol for its promotion of sociability and conviviality (Cochrane et al., 2003). Thus, we might expect a subset of “well-adjusted” Chinese adolescents to report experiences of lifetime substance use, as was observed in the current study. Similarly, in both Indonesia and Malawi, it is possible that being teased or called names is part of the habitual juvenile interactions between youth, and therefore does not always carry the serious consequences associated with more severe forms of bullying (Kubwalo et al., 2013; Yusuf et al., 2019). It is also possible that some of the nuances within the latent classes relate to important contextual differences between samples: for example, we would hypothesize that greater overall economic development within Shanghai may facilitate increased access to alcohol among youth.

Finally, through tests of measurement invariance, we found that both the prevalence and nature of psychosocial risk classes differed significantly by sex within each country, emphasizing the importance of explicitly testing for measurement invariance by sex within person-centered analyses rather than simply controlling for sex as a covariate. While this is not a surprising finding given the well-established differences in emotional and behavioral challenges between boys and girls in this age group (Zahn-Waxler et al., 2015), some of the specific results run counter to our expectations. In particular, there was little consistency across countries in terms of which indicators exhibited sex-specific invariance: in Malawi, there was invariance in the emotional but not behavioral indicators, whereas in DRC, Indonesia, and China, there was invariance in a divergent set of emotional and behavioral indicators. While gender norms are greatly influenced by cultural environments, normative values around masculinity in many settings encourage the adoption of behaviors such as interpersonal violence and substance use (Ragonese et al., 2019). These gender norms often crystalize in early adolescence (Chandra-Mouli et al., 2018; Hill & Lynch, 1983), and may help explain the outsized prevalence of behavioral issues among adolescent boys (Patton et al., 2018). In the context of the current study, we might expect such underlying gender norms to manifest through measurement noninvariance of behavioral indicators, as was observed in Malawi: this would indicate that boys and girls with equivalent levels of underlying psychosocial risk were outwardly expressing this risk in different ways. Our findings, however, tell a more nuanced story: besides *being slapped/hit/hurt*, no single psychosocial indicator demonstrated noninvariance across countries. This suggests a complex relationship in the translation of gender norms into behaviors that defies simple explanations or interventions (Courtenay, 2000). It may also speak to important differences in gender socialization processes across the included countries.

The sex-specific models in each country also suggested that there may be especially heightened vulnerability among boys in this age group. Boys were more likely to be in the *Maladjusted* class across countries, with class prevalences of up to 6% higher than their female counterparts. Further, boys within this subgroup in DRC and Indonesia had a higher likelihood of endorsing a number of emotional and behavioral indicators (i.e., *worrying* and *feeling sad* in DRC; *sleeplessness*, *bullying/threatening*, and *substance use* in Indonesia). These findings align with an emerging body of research which suggests that adolescent boys and young men face disproportionately high mental health challenges compared to their female peers (Rice et al., 2018). These challenges are thought to stem from a confluence of related factors – including a greater disconnection from health services, the stigmatization of emotional vulnerability, and a lack of recognition of masculine variants of distress – and ultimately contribute to elevated rates of violence, substance abuse, suicide, and premature death among men throughout the life course (Bell et al., 2013; Cavanagh et al., 2017; Rice et al., 2018). While global health and development policies have historically focused on girls and young women due to the stark disadvantages that they face worldwide (Baker et al., 2014; Hawkes & Buse, 2013), such findings have led a number of researchers to call for greater gender sensitivity in programs targeting mental health and well-being among young people (Amin et al., 2018; Gwyther et al., 2019; Rice et al., 2018). Our results lend further support to such calls to action, as they suggest that early adolescent boys may experience particular psychosocial risks that should be addressed through intervention activities.

Together, our findings have important implications for preventive interventions targeting psychosocial adjustment among early adolescents in LMICs. First, the heterogeneous nature of the latent classes speaks to the incompatibility of a “one-size-fits-all approach” for addressing psychosocial risks among this age group. Instead, it is clear that across diverse contexts, there is a need for targeted intervention strategies that take into account youth’s distinctive emotional and/or behavioral needs. Second, the marked sex differences within equivalent latent classes are indicative that such approaches should be gender sensitive in order to maximize their impact. Indeed, prior studies of mental health and psychosocial support interventions conducted among adolescents in LMICs have often identified disparate effects for boys and girls (Betancourt et al., 2012; Bolton et al., 2007; Tol et al., 2014): this supports the notion that intervention components may need to be tailored in order to appropriately address unique developmental challenges faced by boys and girls in this age group. In practice, this might mean a mix of gender-specific and combined group activities within intervention programs. Third, the presence of a *Maladjusted* class across countries can be used to guide resource allocation decisions. While prevention strategies would ideally follow a multitiered approach – including some universal interventions targeting all adolescents, and some selected or indicated interventions targeting only those at the greatest risk (O’Connell et al., 2009) – resource limitations in LMICs often make it necessary to prioritize populations deemed to be the most vulnerable. These findings could be used to inform such prioritization, as they suggest that there may be a particular need for services among a small subgroup of adolescents with co-occurring emotional and behavioral problems. Finally, and perhaps most importantly, the consistency in psychosocial risk patterns across diverse country settings suggests that interventions targeting early adolescents living in contexts of adversity may have broad cross-national applicability. Given the dearth of evidence from LMICs (Fazel et al., 2014; Patel et al., 2008), these results support the adaptation and implementation of existing interventions with proven success in reaching vulnerable youth.

Importantly, while the similarity of psychosocial risk patterns across four diverse countries is striking, it does not remove the need for extensive cultural adaptation when considering appropriate youth-focused psychosocial interventions. For example, nuances related to aggressive behavior in China necessitate a targeted examination of how to appropriately address this issue among vulnerable adolescents within Chinese contexts. Likewise, for interventions that seek to take into account the ways in which gender norms influence psychosocial risk, these results emphasize the fact that such norms may operate differently in Malawi compared to DRC, China, or Indonesia. The need for adaptation also arises from within-country contextual differences: for instance, whereas this study included only in-school youth from low-resource urban areas, different strategies may be required to reach adolescents living in rural areas or those who are not actively enrolled in school. In tailoring interventions to meet the needs of adolescents within these countries, subsequent research should attempt to better understand specific cultural drivers underlying cross-national differences in psychosocial risk patterns, as well as the extent to which findings apply to diverse populations.

The current study has several important limitations to consider. Across countries, LCA was used as an exploratory data analysis technique, with researcher judgment factoring heavily into the selection of final models, especially given inconsistencies between various fit indices. While such judgments may make results difficult to replicate, we have documented our methodological decision-making extensively in order to maximize transparency and encourage replication (Collins & Lanza, 2010; Schoot et al., 2017). In addition, the data driven nature of LCA means that identified classes could be sample-specific statistical artifacts rather than naturally occurring subgroups (Bauer & Curran, 2004); however, the consistency of findings across four separate populations, as well as their similarity to those of external researchers (Bianchi et al., 2017; Kretschmer et al., 2015), suggests the reliability and validity of subgroups. While we considered a range of emotional and behavioral problems, there are further indicators that would have been beneficial to include in our analyses in order to better align with prior research. In particular, future studies should focus on the ways in which attention problems coincide with the other included emotional and behavioral indicators as they have been found to be an essential part of the dysregulation profile in other contexts (Althoff et al., 2010; Jordan et al., 2016). While symptoms of depression and anxiety were originally measured using items with Likert-type response scales, these indicators were dichotomized for analysis. Although this approach allowed for simultaneous analysis alongside behavioral indicators, it also resulted in a loss of information. In order to further address this limitation, we performed sensitivity analyses exploring alternative approaches to dichotomization; results did not differ substantively from our initial findings. In addition, all data were assessed by adolescent self-report and are therefore susceptible to social desirability bias, although it is likely that the use of CASI helped to mitigate this problem (Le et al., 2006). The cross-sectional nature of these data also precludes an examination of the stability of these classes over time; given the longitudinal nature of the GEAS, however, there is an opportunity to explore this issue once data become available. Finally, while LCA has important applications for prevention research due to its ability to inform interventions targeting specific subgroups (Nylund-Gibson & Hart, 2014), it should be noted that these methods are not intended to directly screen adolescents into services.

Despite these limitations, this study has a number of notable strengths, including its large sample size, its comparison of early adolescents across four LMICs, its simultaneous analysis of emotional and behavioral problems, and its novel examination of sex-related measurement invariance. Using a person-centered analytic approach, we identified four consistent classes of psychosocial challenges among early adolescents across DRC, Malawi, Indonesia, and China. Tests of measurement invariance indicated there were nuances between boys and girls within equivalent classes, suggesting the importance of gender in shaping the expression of psychosocial risk. Taken together, these findings can be used to support the tailoring of interventions targeting psychosocial adjustment among subgroups of early adolescents with increased vulnerability, and indicate that such programs may have wide utility across diverse cross-national settings.

## Supplementary Material

1

## Figures and Tables

**Table 1.** Adolescent sociodemographic characteristics and psychosocial risks by country

	DRC (*n* = 2006)	Malawi (*n* = 2016)	Indonesia (*n* = 4657)	China (*n* = 1758)
Girls: *N* (%)	1033 (51.5)	999 (49.6)	2469 (53.0)	855 (48.6)
Age: M ± SD	11.9 ± 1.4	12.1 ± 1.1	12.2 ± 0.5	12.5 ± 1.0
Household size: M ± SD	7.3 ± 2.6	5.7 ± 1.9	4.8 ± 1.3	3.7 ± 1.1
Migrated to current city: *N* (%)	291 (14.5)	–	491 (10.5)	263 (15.0)
Primary caregiver: *N* (%)				
Mother	503 (25.1)	1485 (73.7)	3,267 (70.2)	1246 (70.9)
Father	1144 (57.0)	128 (6.4)	1018 (21.9)	256 (14.6)
Grandparent	131 (6.5)	140 (6.9)	74 (1.6)	217 (12.3)
Other	161 (8.0)	252 (12.5)	85 (1.8)	30 (1.7)
Primary caregiver’s marital status: *N* (%)				
Married/living together	942 (47.0)	–	4159 (89.3)	1530 (87.0)
Unmarried/separated/widowed	996 (49.7)	–	272 (5.8)	190 (10.8)
Primary caregiver’s education: *N* (%)				
Primary school or less	118 (5.9)	–	631 (13.6)	78 (4.4)
Some or all secondary school	1045 (52.1)	–	2309 (49.6)	340 (19.3)
Some or all vocational school or university	742 (37.0)	–	1493 (32.1)	1115 (63.4)
Primary caregiver’s employment status: *N* (%)				
Employed/retired	1516 (75.6)	–	2701 (58.0)	1495 (85.0)
Unemployed	423 (21.1)	–	1,601 (34.4)	199 (11.3)
Emotional problems: *N* (%)				
Blame myself when things go wrong	1419 (70.7)	1485 (73.7)	2830 (60.8)	1322 (75.2)
Worry for no good reason	274 (13.7)	1131 (56.1)	2275 (48.9)	749 (42.6)
So unhappy I can’t sleep at night	288 (14.4)	804 (39.9)	1435 (30.8)	502 (28.6)
Feel sad	360 (18.0)	1213 (60.2)	1419 (30.5)	510 (29.0)
So unhappy I think of self-harm	95 (4.7)	597 (29.6)	924 (19.8)	285 (16.2)
Behavioral problems: *N* (%)				
Bullied/threatened	489 (24.4)	485 (24.1)	517 (11.1)	81 (4.6)
Slapped/hit/physically hurt	493 (24.6)	583 (28.9)	581 (12.5)	77 (4.4)
Been teased/called names	723 (36.0)	1062 (52.7)	2253 (48.4)	559 (31.8)
Been slapped/hit/physically hurt	450 (22.4)	823 (40.8)	751 (16.1)	240 (13.7)
Used substance	208 (10.4)	421 (20.9)	520 (11.2)	474 (27.0)

*Note*. In DRC and Indonesia, household size, primary caregiver, primary caregiver’s marital status, primary caregiver’s education, and primary caregiver’s employment status are based on caregiver-reported data. In Malawi, migration status, primary caregiver’s marital status, primary caregiver’s education, and primary caregiver’s employment status are not reported.

**Table 2.** Latent class analysis fit statistics by country

Number of classes	LL	AIC	BIC	aBIC	VLMR	Entropy
*DRC (n = 2006)*						
1	−9425.06	18,870.13	18,926.17	18,894.40	–	–
2	−8594.14	17,230.27	17,347.95	17,281.24	<0.001	0.80
3	−8439.98	16,943.95	17,123.28	17,021.61	<0.001	0.78
4	−8380.42	16,846.85	**17,087.81**	**16,951.20**	0.001	0.77
5	−8363.11	16,834.23	17,136.84	16,965.28	0.538	0.72
6	−8347.75	16,825.50	17,189.75	16,983.24	0.079	0.64
7	−8332.93	16,817.86	17,243.76	17,002.30	0.084	0.69
*Malawi (n = 2016)*						
1	−12,150.47	24,320.94	24,377.03	24,345.26	–	–
2	−11,209.29	22,460.57	22,578.36	22,511.64	0.002	0.70
3	−10,866.74	21,797.48	21,976.96	21,875.30	<0.001	0.75
4	−10,689.89	21,465.78	21,706.96	21,570.35	<0.001	0.72
5	−10,640.46	21,388.92	**21,691.80**	21,520.23	0.056	0.69
6	−10,604.64	21,339.28	21,703.86	**21,497.35**	0.176	0.69
7	−10,581.73	21,315.46	21,741.74	21,500.28	0.001	0.70
*Indonesia (n = 4657)*						
1	−23,817.74	47,655.48	47,719.95	47,688.17	–	–
2	−21,894.86	43,831.72	43,967.09	43,900.36	<0.001	0.71
3	−21,110.09	42,284.17	42,490.45	42,388.76	<0.001	0.75
4	−20,798.93	41,683.85	41,961.04	41,824.40	<0.001	0.74
5	−20,642.32	41,392.63	41,740.72	41,569.13	0.002	0.70
6	−20,589.85	41,309.71	41,728.71	41,522.16	0.005	0.71
7	−20,539.99	41,231.98	**41,721.89**	**41,480.39**	0.018	0.69
*China (n = 1758)*						
1	−8416.46	16,852.92	16,907.64	16,875.87	–	–
2	−7767.71	15,324.31	15,692.33	15,625.61	<0.001	0.71
3	−7630.16	15,266.84	**15,499.41**	15,397.75	<0.001	0.75
4	−7590.42	15,249.36	15,502.14	**15,365.53**	0.003	0.77
5	−7570.68	15,235.78	15,544.84	15,373.29	0.039	0.75
6	−7552.89	15,324.31	15,591.46	15,384.96	0.006	0.78
7	−7535.20	15,222.40	15,638.26	15,396.82	0.004	0.82

*Note*. LL = log likelihood; AIC = Akaike Information Criteria; BIC = Bayesian Information Criteria; aBIC = sample size-adjusted Bayesian Information Criteria; VLMR = Vuong-Lo-Mendell-Rubin Likelihood Ratio Test. VLMR based on latent class models without clustered standard errors. Bold indicates best-fitting model as suggested by the BIC and aBIC.

**Table 3.** Model comparisons for measurement invariance testing by sex for the multigroup models in each country

Model	Description	LL	Npar	SC	Comparison	df	LRTS	* p* value
*DRC*								
1.0	Fully unconstrained	−9687.62	87	1.10	–	–	–	–
1.1	Item-response probabilities constrained	−9751.44	47	1.12	1.1 vs. 1.0	40	119.37	<0.001
2.1	Self-blame (1) constrained	−9695.10	83	1.11	2.1 vs. 1.0	4	18.84	0.001
2.2	Worry (2) constrained	−9692.35	83	1.11	2.2 vs. 1.0	4	11.36	0.023
2.3	Can’t sleep (3) constrained	−9693.01	83	1.07	2.3 vs. 1.0	4	6.20	0.185
2.4	Feel sad (4) constrained	−9692.26	83	1.11	2.4 vs. 1.0	4	10.40	0.034
2.5	Think of self-harm (5) constrained	−9689.02	83	1.10	2.5 vs. 1.0	4	2.73	0.605
2.6	Bullied/threatened (6) constrained	−9690.00	83	1.12	2.6 vs. 1.0	4	8.55	0.073
2.7	Slapped/hit/hurt (7) constrained	−9690.39	83	1.11	2.7 vs. 1.0	4	7.06	0.132
2.8	Been teased/called names (8) constrained	−9698.87	83	1.15	2.8 vs. 1.0	4	214.67	<0.001
2.9	Been slapped/hit/hurt (9) constrained	−9703.14	83	1.10	2.9 vs. 1.0	4	28.36	<0.001
2.10	Substance use (10) constrained	−9691.51	83	1.11	2.10 vs. 1.0	4	9.20	0.056
3.1	Indicators 3 and 5 constrained	−9693.23	79	1.07	3.1 vs. 1.0	8	0.442	7.91
3.2	Indicators 3, 5, and 6 constrained	−9695.95	75	1.09	3.2 vs. 1.0	12	14.55	0.267
3.3	Indicators 3, 5, 6, and 7 constrained	−9698.19	71	1.08	3.3 vs. 1.0	16	17.88	0.331
**3.4**	**Indicators 3, 5, 6, 7, and 10 constrained**	−**9700.95**	**67**	**1.09**	**3.4 vs. 1.0**	20	**23.89**	**0.247**
4.1	Class prevalences constrained	−9713.69	64	1.14	5.1 vs. 3.4	3	148.13	<0.001
*Malawi*								
1.0	Fully unconstrained	−12,031.75	87	1.17	–	–	–	–
1.1	Item-response probabilities constrained	−12,076.94	47	1.42	1.1 vs. 1.0	40	102.36	<0.001
2.1	Self-blame (1) constrained	−12,036.33	83	1.21	2.1 vs. 1.0	4	22.90	<0.001
2.2	Worry (2) constrained	−12,035.79	83	1.22	2.2 vs. 1.0	4	50.67	<0.001
2.3	Can’t sleep (3) constrained	−12,034.79	83	1.23	2.3 vs. 1.0	4	331.57	<0.001
2.4	Feel sad (4) constrained	−12,033.10	83	1.17	2.4 vs. 1.0	4	2.16	0.706
2.5	Think of self-harm (5) constrained	−12,032.67	83	1.16	2.5 vs. 1.0	4	1.20	0.879
2.6	Bullied/threatened (6) constrained	−12,040.52	83	1.28	2.6 vs. 1.0	4	−16.79	–
2.7	Slapped/hit/hurt (7) constrained	−12,035.80	83	1.24	2.7 vs. 1.0	4	−56.41	–
2.8	Been teased/called names (8) constrained	−12,038.80	83	1.32	2.8 vs. 1.0	4	−7.57	–
2.9	Been slapped/hit/hurt (9) constrained	−12,037.17	83	1.29	2.9 vs. 1.0	4	−8.72	–
2.10	Substance use (10) constrained	−12,051.49	83	1.41	2.10 vs. 1.0	4	−10.59	–
3.1	Indicators 4 and 5 constrained	−12,033.83	79	1.17	3.1 vs. 1.0	8	3.29	0.915
3.2	Indicators 3, 4, and 5 constrained	−12,038.33	75	1.22	3.2 vs. 1.0	12	15.06	0.238
3.3	Indicators 2, 3, 4, and 5 constrained	−12,042.70	71	1.21	3.3 vs. 1.0	16	21.22	0.170
**3.4**	**Indicators 1, 2, 3, 4, and 5 constrained**	−**12,047.10**	**67**	**1.20**	**3.4 vs. 1.0**	**20**	**28.28**	**0.103**
4.1	Class prevalences constrained	−12,052.26	64	1.26	5.1 vs. 3.4	3	−89.10	–
*Indonesia*								
1.0	Fully unconstrained	−23,636.94	87	1.60	–	–	–	–
1.1	Item-response probabilities constrained	−23,871.07	47	1.93	1.1 vs. 1.0	40	386.82	<0.001
2.1	Self-blame (1) constrained	−23,640.23	83	1.61	2.1 vs. 1.0	4	4.71	0.318
2.2	Worry (2) constrained	−23,639.40	83	1.61	2.2 vs. 1.0	4	3.46	0.485
2.3	Can’t sleep (3) constrained	−23,653.48	83	1.65	2.3 vs. 1.0	4	62.36	<0.001
2.4	Feel sad (4) constrained	−23,640.24	83	1.62	2.4 vs. 1.0	4	5.88	0.209
2.5	Think of self-harm (5) constrained	−23,653.61	83	1.63	2.5 vs. 1.0	4	33.93	<0.001
2.6	Bullied/threatened (6) constrained	−23,647.95	83	1.60	2.6 vs. 1.0	4	14.43	0.006
2.7	Slapped/hit/hurt (7) constrained	−23,639.87	83	1.58	2.7 vs. 1.0	4	2.93	0.570
2.8	Been teased/called names (8) constrained	−23,642.67	83	1.61	2.8 vs. 1.0	4	8.32	0.080
2.9	Been slapped/hit/hurt (9) constrained	−23,658.37	83	1.62	2.9 vs. 1.0	4	36.72	<0.001
2.10	Substance use (10) constrained	−23,784.29	83	1.72	2.10 vs. 1.0	4	−346.95	–
3.1	Indicators 1 and 2 constrained	−23,644.50	79	1.62	3.1 vs. 1.0	8	11.14	0.194
3.2	Indicators 1, 2, and 4 constrained	−23,648.42	75	1.65	3.2 vs. 1.0	12	17.99	0.116
3.3	Indicators 1, 2, 4, and 7 constrained	−23,650.98	71	1.61	3.3 vs. 1.0	16	18.18	0.314
**3.4**	**Items 1, 2, 4, 7, and 8 constrained**	−**23,659.91**	**67**	**1.64**	**3.4 vs. 1.0**	**20**	**31.15**	**0.053**
4.1	Class prevalences constrained	−23,710.47	64	1.65	5.1 vs. 3.4	3	73.95	<0.001
*China*								
1.0	Fully unconstrained	−8783.53	65	1.22	–	–	–	–
1.1	Item-response probabilities constrained	−8826.64	35	1.33	1.1 vs. 1.0	30	78.86	<0.001
2.1	Self-blame (1) constrained	−8784.12	62	1.24	2.1 vs. 1.0	3	1.59	0.661
2.2	Worry (2) constrained	−8787.22	62	1.28	2.2 vs. 1.0	3	−105.30	–
2.3	Can’t sleep (3) constrained	−8784.22	62	1.26	2.3 vs. 1.0	3	3.24	0.356
2.4	Feel sad (4) constrained	−8785.16	62	1.26	2.4 vs. 1.0	3	9.48	0.024
2.5	Think of self-harm (5) constrained	−8791.79	62	1.29	2.5 vs. 1.0	3	−86.04	–
2.6	Bullied/threatened (6) constrained	−8784.19	62	1.25	2.6 vs. 1.0	3	1.98	0.576
2.7	Slapped/hit/hurt (7) constrained	−8788.86	62	1.13	2.7 vs. 1.0	3	3.49	0.322
2.8	Been teased/called names (8) constrained	−8790.78	62	1.24	2.8 vs. 1.0	3	16.74	0.001
2.9	Been slapped/hit/hurt (9) constrained	−8798.40	62	1.26	2.9 vs. 1.0	3	80.39	<0.001
2.10	Substance use (10) constrained	−8784.00	62	1.17	2.10 vs. 1.0	3	0.43	0.934
3.1	Indicators 1 and 3 constrained	−8784.77	59	1.30	3.1 vs. 1.0	6	5.28	0.509
3.2	Indicators 1, 3, and 6 constrained	−8785.56	56	1.32	3.2 vs. 1.0	9	6.56	0.683
3.3	Indicators 1, 3, 6, and 7 constrained	−8792.75	53	1.21	3.3 vs. 1.0	12	14.75	0.255
3.4	Indicators 1, 3, 6, 7, and 10 constrained	−8793.17	50	1.15	3.4 vs. 1.0	15	13.44	0.569
3.5	Indicators 1, 3, 4, 6, 7, and 10 constrained	−8794.72	47	1.20	3.5 vs. 1.0	18	17.54	0.486
**3.6**	**Indicators 1, 2, 3, 4, 6, 7, and 10 constrained**	−**8798.51**	**44**	**1.25**	**3.6 vs. 1.0**	**21**	**25.72**	**0.217**
4.1	Class prevalences constrained	−8818.20	42	1.13	4.1 vs. 3.6	2	10.46	0.005

*Note*. LL = log likelihood; Npar = number of parameters; SC = scaling correction factor; LRTS = likelihood ratio test statistic. Bold indicates best-fitting model as suggested by the LRTS. No *p* values reported for model comparisons with a negative LRTS.
