# Supplementary material for: A latent class approach to understanding patterns of emotional and behavioral problems among early adolescents across four low- and middle-income countries
Source: Dev Psychopathol. Author manuscript; Available in PMC 2024 Apr 1. (PMC9708939; doi:10.1017/S0954579422000384)
Supplement: 1 [file NIHMS1791399-supplement-1.docx]

**Supplemental Tables**

**Supplemental Table 1.** Latent class analysis fit statistics for Indonesian study sites

| **Number of classes** | **LL** | **AIC** | **BIC** | **aBIC** | **VLMR** | **Entropy** |
| --- | --- | --- | --- | --- | --- | --- |
| *Semarang (n = 1,517)* |  |  |  |  |  |  |
| 1 | -7790.06 | 15600.11 | 15653.36 | 15621.59 | - | - |
| 2 | -7322.81 | 14687.61 | 14799.42 | 14732.71 | 0.011 | 0.66 |
| 3 | -7107.29 | 14278.58 | 14448.97 | 14347.31 | <0.001 | 0.72 |
| 4 | -7023.37 | 14132.74 | **14361.70** | 14225.10 | 0.396 | 0.67 |
| 5 | -6983.82 | 14075.64 | 14363.17 | 14191.62 | <0.001 | 0.70 |
| 6 | -6959.36 | 14048.72 | 14394.81 | **14188.32** | 0.387 | 0.69 |
| 7 | -6944.38 | 14040.75 | 14445.41 | 14203.98 | 0.554 | 0.70 |
| *Bandar Lampung (n = 1,391)* |  |  |  |  |  |  |
| 1 | -7083.36 | 14186.72 | 14239.10 | 14207.34 | - | - |
| 2 | -6338.14 | 12718.29 | 12828.28 | 12761.57 | <0.001 | 0.74 |
| 3 | -6017.11 | 12098.23 | 12265.84 | 12164.19 | <0.001 | 0.78 |
| 4 | -5920.27 | 11926.54 | 12151.77 | 12015.17 | 0.056 | 0.78 |
| 5 | -5877.61 | 11863.23 | **12146.07** | 11974.53 | 0.106 | 0.69 |
| 6 | -5848.85 | 11827.71 | 12168.16 | **11961.68** | 0.165 | 0.68 |
| 7 | -5827.59 | 11807.18 | 12205.26 | 11963.83 | 0.449 | 0.68 |
| *Denpasar (n = 1,749)* |  |  |  |  |  |  |
| 1 | -8827.43 | 17674.87 | 17729.53 | 17697.76 | - | - |
| 2 | -8088.38 | 16218.75 | 16333.55 | 16266.84 | <0.001 | 0.72 |
| 3 | -7809.30 | 15682.59 | 15857.53 | 15755.87 | <0.001 | 0.77 |
| 4 | -7698.90 | 15483.81 | 15718.88 | 15582.27 | <0.001 | 0.76 |
| 5 | -7617.20 | 15342.39 | **15637.60** | **15466.04** | <0.001 | 0.73 |
| 6 | -7594.85 | 15319.70 | 15675.04 | 15468.55 | 0.129 | 0.74 |
| 7 | -7581.65 | 15315.30 | 15730.78 | 15489.34 | 0.336 | 0.74 |

*Note.* LL = log likelihood; AIC = Akaike Information Criteria; BIC = Bayesian Information Criteria; aBIC = sample size-adjusted Bayesian Information Criteria; VLMR = Vuong-Lo-Mendell-Rubin Likelihood Ratio Test. VLMR based on latent class models without clustered standard errors. Bold indicates best-fitting model as suggested by the BIC and aBIC.

**Supplemental Table 2.** Latent class analysis fit statistics by country and sex

| **Number of classes** | **LL** | **AIC** | **BIC** | **aBIC** | **VLMR** | **Entropy** |
| --- | --- | --- | --- | --- | --- | --- |
| *DRC boys (n = 973)* |  |  |  |  |  |  |
| 1 | -4799.64 | 9619.28 | 9668.08 | 9636.32 | - | - |
| 2 | -4339.86 | 8721.72 | 8824.21 | 8757.51 | <0.001 | 0.81 |
| 3 | -4257.18 | 8578.35 | 8734.52 | 8632.89 | <0.001 | 0.79 |
| 4 | -4216.81 | 8519.61 | **8729.47** | 8592.90 | 0.006 | 0.79 |
| 5 | -4196.38 | 8500.75 | 8764.29 | **8592.79** | 0.436 | 0.74 |
| 6 | -4183.26 | 8496.51 | 8813.74 | 8607.30 | 0.020 | 0.76 |
| *DRC girls (n = 1,033)* |  |  |  |  |  |  |
| 1 | -4541.63 | 9103.25 | 9152.657 | 9120.90 | - | - |
| 2 | -4190.74 | 8423.49 | 8527.23 | 8460.53 | <0.001 | 0.79 |
| 3 | -4108.60 | 8281.20 | **8439.29** | 8337.65 | 0.001 | 0.78 |
| 4 | -4081.26 | 8248.52 | 8460.95 | **8324.38** | 0.041 | 0.77 |
| 5 | -4066.05 | 8240.09 | 8506.86 | 8335.35 | 0.366 | 0.67 |
| 6 | -4053.02 | 8236.04 | 8557.15 | 8350.71 | 0.129 | 0.71 |
| *Malawi boys (n = 1,017)* |  |  |  |  |  |  |
| 1 | -6270.22 | 12560.44 | 12609.69 | 12577.93 | - | - |
| 2 | -5778.59 | 11599.18 | 11702.59 | 11635.90 | <0.001 | 0.71 |
| 3 | -5593.28 | 11250.55 | 11408.14 | 11306.50 | <0.001 | 0.78 |
| 4 | -5509.46 | 11104.92 | **11316.68** | 11180.10 | <0.001 | 0.75 |
| 5 | -5478.36 | 11064.71 | 11330.64 | **11159.13** | 0.228 | 0.70 |
| 6 | -5457.84 | 11045.68 | 11365.77 | 11159.33 | 0.134 | 0.74 |
| *Malawi girls (n = 999)* |  |  |  |  |  |  |
| 1 | -5837.54 | 11695.08 | 11744.14 | 11712.38 | - | - |
| 2 | -5371.33 | 10784.65 | 10887.69 | 10821.00 | <0.001 | 0.72 |
| 3 | -5212.98 | 10489.95 | 10646.97 | 10545.33 | <0.001 | 0.74 |
| 4 | -5124.99 | 10335.98 | **10546.97** | 10410.40 | <0.001 | 0.73 |
| 5 | -5103.63 | 10315.25 | 10580.21 | 10408.71 | 0.512 | 0.71 |
| 6 | -5081.81 | 10293.62 | 10612.56 | **10406.12** | 0.326 | 0.68 |
| *Indonesia boys (n = 2,188)* |  |  |  |  |  |  |
| 1 | -12199.11 | 24418.22 | 24475.13 | 24443.35 | - | - |
| 2 | -11152.69 | 22347.38 | 22466.88 | 22400.16 | <0.001 | 0.76 |
| 3 | -10723.46 | 21510.92 | 21693.03 | 21591.36 | <0.001 | 0.78 |
| 4 | -10519.55 | 21125.10 | 21369.80 | 21233.18 | <0.001 | 0.75 |
| 5 | -10434.78 | 20977.56 | **21284.86** | 21113.30 | 0.151 | 0.72 |
| 6 | -10395.42 | 20920.84 | 21290.74 | **21084.22** | <0.001 | 0.75 |
| *Indonesia girls (n = 2,469)* |  |  |  |  |  |  |
| 1 | -11097.23 | 22214.46 | 22272.58 | 22240.81 | - | - |
| 2 | -10263.89 | 20569.78 | 20691.82 | 20625.10 | <0.001 | 0.68 |
| 3 | -9995.88 | 20055.75 | 20241.72 | 20140.05 | <0.001 | 0.72 |
| 4 | -9897.89 | 19881.77 | 20131.67 | 19995.05 | 0.012 | 0.73 |
| 5 | -9820.55 | 19749.09 | **20062.92** | 19891.34 | <0.001 | 0.68 |
| 6 | -9792.63 | 19715.27 | 20093.02 | **19886.50** | 0.135 | 0.69 |
| *China boys (n = 903)* |  |  |  |  |  |  |
| 1 | -4409.80 | 8839.60 | 8887.66 | 8855.90 | - | - |
| 2 | -4068.81 | 8179.62 | 8280.54 | 8213.84 | <0.001 | 0.71 |
| 3 | -3990.05 | 8044.09 | **8197.87** | 8096.25 | <0.001 | 0.77 |
| 4 | -3960.37 | 8006.74 | 8213.38 | **8076.82** | 0.021 | 0.77 |
| 5 | -3946.37 | 8000.74 | 8260.25 | 8088.75 | 0.145 | 0.75 |
| 6 | -3933.98 | 7997.97 | 8310.34 | 8103.91 | 0.718 | 0.75 |
| *China girls (n = 855)* |  |  |  |  |  |  |
| 1 | -3950.17 | 7920.34 | 7967.85 | 7936.10 | - | - |
| 2 | -3631.24 | 7304.48 | 7404.25 | 7337.56 | <0.001 | 0.72 |
| 3 | -3575.59 | 7215.17 | **7367.21** | **7265.58** | <0.001 | 0.75 |
| 4 | -3561.02 | 7208.03 | 7412.33 | 7275.77 | 0.463 | 0.65 |
| 5 | -3545.96 | 7199.92 | 7456.48 | 7284.99 | 0.316 | 0.78 |
| 6 | -3533.20 | 7196.40 | 7505.22 | 7298.80 | 0.196 | 0.77 |

*Note:* LL = log likelihood; AIC = Akaike Information Criteria; BIC = Bayesian Information Criteria; aBIC = sample size-adjusted Bayesian Information Criteria; VLMR = Vuong-Lo-Mendell-Rubin Likelihood Ratio Test. VLMR based on latent class models without clustered standard errors. Bold indicates best-fitting model as suggested by the BIC and aBIC.

**Supplemental Table 3.** Adolescent psychosocial problems by country and sex

|  | **DRC** | | | **Malawi** | | | **Indonesia** | | | **China** | | |
| --- | --- | --- | --- | --- | --- | --- | --- | --- | --- | --- | --- | --- |
|  | Boys  *n* = 973 | Girls  *n* = 1,033 | *p* value | Boys  *n* = 1,017 | Girls  *n* = 999 | *p* value | Boys  *n* = 2,188 | Girls  *n* = 2,469 | *p* value | Boys  *n* = 903 | Girls  *n* = 855 | *p* value |
| Emotional problems: N (%) |  |  |  |  |  |  |  |  |  |  |  |  |
| Blame myself when things go wrong | **737**  **(75.8)** | **682**  **(66.0)** | **<0.001** | 749  (73.7) | 736  (73.7) | 0.717 | 1,350  (61.7) | 1,470  (59.9) | 0.108 | 675  (74.8) | 647  (75.7) | 0.650 |
| Worry for no good reason | **155 (15.9)** | **119 (11.5)** | **0.004** | 557  (54.8) | 574  (57.5) | 0.090 | 1,037  (47.4) | 1,238  (50.1) | 0.064 | **355**  **(39.3)** | **394**  **(46.1)** | **0.005** |
| So unhappy I can't sleep at night | **162**  **(16.7)** | **126**  **(12.2)** | **0.005** | 408  (40.1) | 396  (39.6) | 0.746 | **768**  **(35.1)** | **667**  **(27.0)** | **<0.001** | 239  (26.5) | 263  (30.8) | 0.053 |
| Feel sad | 159  (16.3) | 201  (19.5) | 0.071 | 596  (58.6) | 617  (61.8) | 0.298 | 659  (30.1) | 760  (30.8) | 0.797 | **240**  **(26.6)** | **270**  **(31.6)** | **0.024** |
| So unhappy I think of self-harm | 52  (5.3) | 43  (4.2) | 0.216 | 284  (27.9) | 313  (31.3) | 0.080 | **531**  **(24.3)** | **393**  **(15.9)** | **<0.001** | 137  (15.2) | 148  (17.3) | 0.240 |
| Behavioral problems: N (%) |  |  |  |  |  |  |  |  |  |  |  |  |
| Bullied/threatened | **275**  **(28.3)** | **214**  **(20.7)** | **<0.001** | **289**  **(28.4)** | **196**  **(19.6)** | **<0.001** | **367**  **(16.8)** | **150**  **(6.1)** | **<0.001** | 44  (4.9) | 37  (4.3) | 0.490 |
| Slapped/hit/physically hurt | **273**  **(28.1)** | **220**  **(21.3)** | **<0.001** | **316**  **(31.1)** | **267**  **(26.7)** | **0.044** | **372**  **(17.0)** | **209**  **(8.5)** | **<0.001** | **59**  **(6.5)** | **19**  **(2.1)** | **<0.001** |
| Been teased/called names | **427**  **(43.9)** | **296**  **(28.7)** | **<0.001** | **577**  **(56.7)** | **485**  **(48.6)** | **0.001** | **1,114**  **(50.9)** | **1,139**  **(46.1)** | **<0.001** | **320**  **(35.4)** | **239**  **(28.0)** | **<0.001** |
| Been slapped/ hit/physically hurt | **275 (28.3)** | **175**  **(16.9)** | **<0.001** | **444**  **(43.7)** | **379**  **(37.9)** | **0.014** | **527**  **(24.1)** | **224**  **(9.1)** | **<0.001** | **175**  **(19.4)** | **65**  **(7.6)** | **<0.001** |
| Used substance | **121**  **(12.4)** | **87**  **(8.4)** | **0.003** | **268**  **(26.4)** | **153**  **(15.3)** | **<0.001** | **461**  **(21.1)** | **59**  **(2.4)** | **<0.001** | 245  (27.1) | 229  (26.8) | 0.676 |

**Supplemental Table 4.** Estimated class prevalence and item-response probabilities from the latent class models in each country

|  | **DRC (*n* = 2,006)** | | | | **Malawi (*n* = 2,016)** | | | | **Indonesia (*n* = 4,657)** | | | | **China (*n* = 1,758)** | | |
| --- | --- | --- | --- | --- | --- | --- | --- | --- | --- | --- | --- | --- | --- | --- | --- |
|  | WA  (60%) | EP  (14%) | BP  (22%) | MA  (4%) | WA  (40%) | EP  (24%) | BP  (21%) | MA  (15%) | WA  (49%) | EP  (29%) | BP  (15%) | MA  (6%) | WA  (62%) | EP  (28%) | MA  (10%) |
| Emotional problems: |  |  |  |  |  |  |  |  |  |  |  |  |  |  |  |
| Blame myself when things go wrong | **0.67** | **0.87** | **0.69** | **0.81** | **0.67** | **0.90** | **0.66** | **0.94** | 0.46 | **0.89** | **0.51** | **0.92** | **0.67** | **0.94** | **0.77** |
| Worry for no good reason | 0.07 | 0.42 | 0.09 | 0.44 | 0.35 | **0.89** | 0.42 | **0.95** | 0.28 | **0.86** | 0.36 | **0.94** | 0.21 | **0.84** | **0.66** |
| So unhappy I can't sleep at night | 0.03 | **0.55** | 0.07 | **0.69** | 0.11 | **0.82** | 0.18 | **0.90** | 0.09 | **0.66** | 0.19 | **0.86** | 0.08 | **0.66** | **0.52** |
| Feel sad | 0.08 | **0.63** | 0.11 | **0.50** | 0.38 | **0.96** | 0.46 | **0.96** | 0.09 | **0.64** | 0.18 | **0.86** | 0.08 | **0.68** | 0.49 |
| So unhappy I think of self-harm | 0.01 | 0.16 | 0.02 | 0.33 | 0.07 | **0.57** | 0.16 | **0.74** | 0.02 | 0.42 | 0.12 | **0.84** | 0.03 | 0.39 | 0.38 |
| Behavioral problems: |  |  |  |  |  |  |  |  |  |  |  |  |  |  |  |
| Bullied/threatened | 0.04 | 0.17 | **0.74** | **0.90** | 0.03 | 0.12 | 0.48 | **0.72** | 0.01 | 0.04 | 0.43 | **0.81** | 0.01 | 0.02 | 0.39 |
| Slapped/hit/physically hurt | 0.06 | 0.13 | **0.71** | **0.96** | 0.04 | 0.08 | **0.64** | **0.86** | 0.02 | 0.03 | **0.51** | **0.76** | 0.01 | 0.00 | 0.42 |
| Been teased/called names | 0.16 | 0.38 | **0.79** | **0.92** | 0.27 | **0.53** | **0.84** | **0.90** | 0.36 | **0.56** | **0.90** | **0.90** | 0.21 | 0.48 | **0.90** |
| Been slapped/hit/physically hurt | 0.05 | 0.19 | **0.59** | **0.94** | 0.12 | 0.32 | **0.79** | **0.88** | 0.01 | 0.10 | **0.64** | **0.79** | 0.07 | 0.10 | **0.75** |
| Used substance | 0.05 | 0.11 | 0.20 | 0.31 | 0.09 | 0.18 | 0.31 | 0.43 | 0.05 | 0.08 | 0.26 | 0.44 | 0.22 | 0.33 | **0.50** |

*Note:* WA = Well-Adjusted; EP = Emotional Problems; BP = Behavioral Problems; MA = Maladjusted. Models estimated separately for each study country. Item-response probabilities greater than 0.50 are bolded to highlight distinctions between classes.

**Supplemental Table 5.** Estimated class prevalences and item-response probabilities from the fully unconstrained multi-group latent class models in each country

| **DRC** | **Boys (*n* = 973)** | | | | **Girls (*n* = 1,033)** | | | |
| --- | --- | --- | --- | --- | --- | --- | --- | --- |
|  | WA  (54%) | EP  (15%) | BP  (26%) | MA  (3%) | WA  (63%) | EP  (15%) | BP  (17%) | MA  (5%) |
| Blame myself when things go wrong | **0.74** | **0.86** | **0.73** | **0.89** | **0.62** | **0.87** | **0.60** | **0.79** |
| Worry for no good reason | 0.08 | 0.44 | 0.13 | **0.53** | 0.05 | 0.38 | 0.06 | 0.30 |
| So unhappy I can't sleep at night | 0.03 | **0.60** | 0.13 | **0.88** | 0.04 | 0.48 | 0.00 | **0.56** |
| Feel sad | 0.04 | **0.58** | 0.12 | **0.82** | 0.10 | **0.69** | 0.11 | 0.30 |
| So unhappy I think of self-harm | 0.02 | 0.17 | 0.03 | 0.42 | 0.01 | 0.14 | 0.02 | 0.29 |
| Bullied/threatened | 0.05 | 0.18 | **0.75** | **1.00** | 0.03 | 0.14 | **0.73** | **0.84** |
| Slapped/hit/physically hurt | 0.06 | 0.13 | **0.76** | **0.92** | 0.06 | 0.13 | **0.63** | **1.00** |
| Been teased/called names | 0.24 | 0.40 | **0.83** | **0.94** | 0.10 | 0.36 | **0.75** | **0.82** |
| Been slapped/hit/physically hurt | 0.06 | 0.14 | **0.75** | **0.97** | 0.04 | 0.21 | 0.41 | **0.81** |
| Used substance | 0.07 | 0.14 | 0.20 | 0.32 | 0.04 | 0.08 | 0.16 | 0.36 |
| **Malawi** | **Boys (*n* = 1,017)** | | | | **Girls (*n* = 999)** | | | |
|  | WA  (44%) | EP  (21%) | BP  (19%) | MA  (16%) | WA  (38%) | EP  (27%) | BP  (21%) | MA  (14%) |
| Blame myself when things go wrong | **0.64** | **0.94** | **0.66** | **0.95** | **0.71** | **0.85** | **0.65** | **0.94** |
| Worry for no good reason | 0.32 | **0.88** | 0.40 | **1.00** | 0.37 | **0.88** | 0.46 | **0.90** |
| So unhappy I can't sleep at night | 0.13 | **0.83** | 0.22 | **0.87** | 0.08 | **0.83** | 0.14 | **0.93** |
| Feel sad | 0.40 | **0.95** | 0.44 | **0.94** | 0.36 | **0.95** | 0.49 | **0.98** |
| So unhappy I think of self-harm | 0.07 | **0.54** | 0.16 | **0.72** | 0.06 | **0.59** | 0.19 | **0.77** |
| Bullied/threatened | 0.06 | 0.17 | **0.62** | **0.72** | 0.02 | 0.10 | 0.37 | **0.70** |
| Slapped/hit/physically hurt | 0.05 | 0.04 | **0.78** | **0.89** | 0.04 | 0.09 | **0.56** | **0.86** |
| Been teased/called names | 0.35 | **0.60** | **0.87** | **0.89** | 0.22 | 0.48 | **0.82** | **0.90** |
| Been slapped/hit/physically hurt | 0.19 | 0.34 | **0.84** | **0.86** | 0.07 | 0.30 | **0.77** | **0.90** |
| Used substance | 0.12 | 0.25 | 0.46 | 0.45 | 0.06 | 0.14 | 0.20 | 0.36 |
| **Indonesia** | **Boys (*n* = 2,188)** | | | | **Girls (*n* = 2,469)** | | | |
|  | WA  (47%) | EP  (24%) | BP  (21%) | MA  (8%) | WA  (52%) | EP  (33%) | BP  (10%) | MA  (5%) |
| Blame myself when things go wrong | 0.48 | **0.91** | **0.55** | **0.90** | 0.44 | **0.88** | 0.46 | **0.95** |
| Worry for no good reason | 0.26 | **0.88** | 0.35 | **0.98** | 0.28 | **0.85** | 0.43 | **0.92** |
| So unhappy I can't sleep at night | 0.12 | **0.79** | 0.21 | **0.90** | 0.07 | **0.56** | 0.18 | **0.81** |
| Feel sad | 0.08 | **0.68** | 0.17 | **0.89** | 0.09 | **0.61** | 0.25 | **0.86** |
| So unhappy I think of self-harm | 0.03 | **0.55** | 0.15 | **0.86** | 0.02 | 0.32 | 0.11 | **0.82** |
| Bullied/threatened | 0.01 | 0.04 | **0.52** | **0.89** | 0.01 | 0.03 | 0.30 | **0.57** |
| Slapped/hit/physically hurt | 0.02 | 0.03 | **0.54** | **0.82** | 0.01 | 0.03 | **0.51** | **0.59** |
| Been teased/called names | 0.40 | **0.51** | **0.91** | **0.88** | 0.33 | **0.57** | **0.90** | **0.97** |
| Been slapped/hit/physically hurt | 0.05 | 0.16 | **0.71** | **0.79** | 0.00 | 0.04 | **0.53** | **0.75** |
| Used substance | 0.12 | 0.19 | 0.36 | **0.52** | 0.01 | 0.01 | 0.08 | 0.18 |
| **China** | **Boys (*n* = 903)** | | | | **Girls (*n* = 855)** | | | |
|  | WA  (65%) | EP  (24%) | BP  (0%) | MA  (11%) | WA  (59%) | EP  (32%) | BP  (0%) | MA  (9%) |
| Blame myself when things go wrong | **0.67** | **0.95** | **-** | **0.75** | **0.66** | **0.93** | **-** | **0.80** |
| Worry for no good reason | 0.20 | **0.81** | **-** | **0.59** | 0.21 | **0.83** | **-** | **0.82** |
| So unhappy I can't sleep at night | 0.08 | **0.65** | **-** | 0.49 | 0.07 | **0.67** | **-** | **0.58** |
| Feel sad | 0.08 | **0.69** | - | 0.44 | 0.08 | **0.67** | - | **0.61** |
| So unhappy I think of self-harm | 0.02 | 0.45 | - | 0.28 | 0.04 | 0.31 | - | **0.58** |
| Bullied/threatened | 0.01 | 0.00 | **-** | 0.41 | 0.01 | 0.01 | **-** | 0.39 |
| Slapped/hit/physically hurt | 0.02 | 0.00 | **-** | **0.55** | 0.01 | 0.00 | **-** | 0.22 |
| Been teased/called names | 0.25 | **0.56** | **-** | **0.90** | 0.17 | 0.40 | **-** | **0.89** |
| Been slapped/hit/physically hurt | 0.10 | 0.22 | **-** | **0.80** | 0.04 | 0.02 | **-** | **0.59** |
| Used substance | 0.22 | 0.35 | **-** | 0.48 | 0.22 | 0.30 | **-** | **0.52** |

*Note:* WA = Well-Adjusted; EP = Emotional Problems; BP = Behavioral Problems; MA = Maladjusted. Multi-group models estimated separately for each study country. Item-response probabilities greater than 0.50 are bolded to highlight distinctions between classes.

**Supplemental Table 6.** Estimated class prevalences and item-response probabilities for the partially invariant multi-group latent class models in each country

| **DRC** | **Boys (*n* = 973)** | | | | **Girls (*n* = 1,033)** | | | |
| --- | --- | --- | --- | --- | --- | --- | --- | --- |
|  | WA  (54%) | EP  (16%) | BP  (25%) | MA  (5%) | WA  (66%) | EP  (12%) | BP  (18%) | MA  (3%) |
| Blame myself when things go wrong | **0.74** | **0.86** | **0.73** | **0.81** | **0.63** | **0.87** | **0.62** | **0.82** |
| Worry for no good reason | 0.08 | 0.42 | 0.10 | **0.50** | 0.06 | 0.42 | 0.08 | 0.35 |
| *So unhappy I can't sleep at night* | 0.04 | **0.55** | 0.08 | **0.70** | 0.04 | **0.55** | 0.08 | **0.70** |
| Feel sad | 0.03 | **0.56** | 0.10 | **0.62** | 0.11 | **0.73** | 0.12 | 0.35 |
| *So unhappy I think of self-harm* | 0.01 | 0.16 | 0.02 | 0.36 | 0.01 | 0.16 | 0.02 | 0.36 |
| *Bullied/threatened* | 0.04 | 0.17 | **0.74** | **0.91** | 0.04 | 0.17 | **0.74** | **0.91** |
| *Slapped/hit/physically hurt* | 0.06 | 0.14 | **0.71** | **0.95** | 0.06 | 0.14 | **0.71** | **0.95** |
| Been teased/called names | 0.23 | 0.40 | **0.82** | **0.95** | 0.11 | 0.36 | **0.77** | **0.87** |
| Been slapped/hit/physically hurt | 0.06 | 0.15 | **0.72** | **0.93** | 0.05 | 0.23 | 0.44 | **0.95** |
| *Used substance* | 0.05 | 0.12 | 0.19 | 0.32 | 0.05 | 0.12 | 0.19 | 0.32 |
| **Malawi** | **Boys (*n* = 1,017)** | | | | **Girls (*n* = 999)** | | | |
|  | WA  (44%) | EP  (21%) | BP  (19%) | MA  (16%) | WA  (39%) | EP  (27%) | BP  (21%) | MA  (14%) |
| *Blame myself when things go wrong* | **0.67** | **0.89** | **0.66** | **0.94** | **0.67** | **0.89** | **0.66** | **0.94** |
| *Worry for no good reason* | 0.35 | **0.89** | 0.44 | **0.95** | 0.35 | **0.89** | 0.44 | **0.95** |
| *So unhappy I can't sleep at night* | 0.11 | **0.83** | 0.18 | **0.91** | 0.11 | **0.83** | 0.18 | **0.91** |
| *Feel sad* | 0.38 | **0.96** | 0.47 | **0.96** | 0.38 | **0.96** | 0.47 | **0.96** |
| *So unhappy I think of self-harm* | 0.07 | **0.57** | 0.17 | **0.75** | 0.07 | **0.57** | 0.17 | **0.75** |
| Bullied/threatened | 0.06 | 0.16 | **0.62** | **0.71** | 0.02 | 0.10 | 0.37 | **0.69** |
| Slapped/hit/physically hurt | 0.05 | 0.04 | **0.79** | **0.86** | 0.05 | 0.09 | **0.56** | **0.85** |
| Been teased/called names | 0.35 | **0.59** | **0.87** | **0.89** | 0.22 | 0.48 | **0.82** | **0.90** |
| Been slapped/hit/physically hurt | 0.19 | 0.33 | **0.84** | **0.85** | 0.07 | 0.31 | **0.77** | **0.90** |
| Used substance | 0.12 | 0.25 | 0.44 | 0.46 | 0.07 | 0.14 | 0.19 | 0.37 |
| **Indonesia** | **Boys (*n* = 2,188)** | | | | **Girls (*n* = 2,469)** | | | |
|  | WA  (45%) | EP  (25%) | BP  (21%) | MA  (9%) | WA  (53%) | EP  (33%) | BP  (10%) | MA  (4%) |
| *Blame myself when things go wrong* | 0.46 | **0.89** | **0.51** | **0.90** | 0.46 | **0.89** | **0.51** | **0.90** |
| *Worry for no good reason* | 0.28 | **0.86** | 0.36 | **0.93** | 0.28 | **0.86** | 0.36 | **0.93** |
| So unhappy I can't sleep at night | 0.11 | **0.78** | 0.19 | **0.89** | 0.07 | **0.58** | 0.17 | **0.76** |
| *Feel sad* | 0.09 | **0.65** | 0.17 | **0.86** | 0.09 | **0.65** | 0.17 | **0.86** |
| So unhappy I think of self-harm | 0.03 | **0.54** | 0.13 | **0.85** | 0.02 | 0.34 | 0.09 | **0.80** |
| Bullied/threatened | 0.01 | 0.04 | 0.49 | **0.88** | 0.01 | 0.04 | 0.30 | **0.67** |
| *Slapped/hit/physically hurt* | 0.02 | 0.03 | **0.52** | **0.78** | 0.02 | 0.03 | **0.52** | **0.78** |
| *Been teased/called names* | 0.36 | **0.56** | **0.90** | **0.91** | 0.36 | **0.56** | **0.90** | **0.91** |
| Been slapped/hit/physically hurt | 0.04 | 0.16 | **0.69** | **0.79** | 0.00 | 0.05 | **0.53** | **0.79** |
| Used substance | 0.11 | 0.19 | 0.35 | **0.52** | 0.01 | 0.01 | 0.07 | 0.21 |
| **China** | **Boys (*n* = 903)** | | | | **Girls (*n* = 855)** | | | |
|  | WA  (65%) | EP  (23%) | BP  (0%) | MA  (13%) | WA  (59%) | EP  (33%) | BP  (0%) | MA  (7%) |
| *Blame myself when things go wrong* | **0.67** | **0.93** | **-** | **0.77** | **0.67** | **0.93** | **-** | **0.77** |
| *Worry for no good reason* | 0.21 | **0.83** | **-** | **0.67** | 0.21 | **0.83** | **-** | **0.67** |
| *So unhappy I can't sleep at night* | 0.08 | **0.66** | **-** | **0.52** | 0.08 | **0.66** | **-** | **0.52** |
| *Feel sad* | 0.08 | **0.68** | - | **0.50** | 0.08 | **0.68** | - | **0.50** |
| So unhappy I think of self-harm | 0.02 | 0.45 | - | 0.29 | 0.04 | 0.33 | - | **0.59** |
| *Bullied/threatened* | 0.01 | 0.02 | **-** | 0.39 | 0.01 | 0.02 | **-** | 0.39 |
| *Slapped/hit/physically hurt* | 0.01 | 0.00 | **-** | 0.41 | 0.01 | 0.00 | **-** | 0.41 |
| Been teased/called names | 0.25 | **0.54** | **-** | **0.89** | 0.17 | 0.42 | **-** | **0.90** |
| Been slapped/hit/physically hurt | 0.11 | 0.18 | **-** | **0.80** | 0.03 | 0.04 | **-** | **0.65** |
| *Used substance* | 0.22 | 0.33 | **-** | **0.50** | 0.22 | 0.33 | **-** | **0.50** |

*Note:* WA = Well-Adjusted; EP = Emotional Problems; BP = Behavioral Problems; MA = Maladjusted. Multi-group models estimated separately for each study country. Italicized items are those that were constrained to be equal for boys and girls. Item-response probabilities greater than 0.50 are bolded to highlight distinctions between classes.
